# Assessment of shear bond strength and microleakage of fissure sealant following enamel deproteinization: An *in vitro* study

**DOI:** 10.4317/jced.56281

**Published:** 2020-03-01

**Authors:** Gokcen-Deniz Bayrak, Elif-Beril Gurdogan-Guler, Yagmur Yildirim, Dilek Ozturk, Senem Selvi-Kuvvetli

**Affiliations:** 1 Department of Pediatric Dentistry, Faculty of Dentistry, Yeditepe University, Istanbul, Turkey; 2 Private Practice in Florida, USA

## Abstract

**Background:**

To evaluate the effect of two different deproteinizing agents (5.25% sodium hypochlorite and Papacarie®) prior to acid etching on the shear bond strength and microleakage of a fissure sealant material.

**Material and Methods:**

A total of 123 extracted noncarious human third molar teeth were randomly divided into three groups for each test: acid etching alone (control) (C), sodium hypochlorite (NaOCl), and Papacarie Duo® (PC). The shear bond strength tests were performed using a universal testing machine, and microleakage was evaluated using a dye penetration method.

**Results:**

The mean shear bond strength values obtained in Group C were significantly lower than those of Group NaOCl and Group PC (*p*=0.0001). There were no significant differences between Group NaOCl and Group PC (*p*>0.05). No statistically significant difference was observed among the groups in terms of microleakage (*p*>0.05).

**Conclusions:**

Papacarie Duo® and 5.25% sodium hypochlorite treatments before etching increased the shear bond strength of the fissure sealant. However, pre-treatment with these agents did not decrease the microleakage between teeth and sealant.

** Key words:**Deproteinization, microleakage, papain, dental seal, shear bond strength.

## Introduction

Pit and fissure sealants are commonly used to prevent caries formation in permanent molars, especially in individuals with a higher risk of dental caries. First permanent molars are highly susceptible to caries during the eruption phase because the enamel is immature and it is difficult for children to clean the erupting tooth (1). Sealants act as a physically protective layer that prevents demineralization of enamel by blocking the interaction of fissure microorganisms and their nutrient substrates (2). The success of pit and fissure sealants depends on factors such as an efficient marginal seal, retention, and resistance to microleakage related to the ability of adhesion between the fissure sealant and the enamel (3,4).

The use of phosphoric acid is a well-accepted, conventional method used to create micro-porosities, which are the key in providing sealant retention (5). However, organic remnants as well as fissure morphology and aprismatic enamel structure can decrease etching ability and thus prevent adequate adhesion. Phosphoric acid does not remove organic matter; therefore, effective etching cannot be achieved sufficiently, resulting in an inadequate etching pattern for bonding (6). Various methods for preparing fissures, such as pumice prophylaxis, bonding agents, lasers, air abrasion, and sodium hypochlorite deproteinization, have been recommended to improve sealant retention (7-10).

Currently, enamel deproteinization seems to be a promising option to optimize material adhesion and retention. Deproteinization removes the surface organic matter layer prior to acid etching, which aids in creating the desired type I or type II etching pattern. A portion of the organic residue present on the enamel surface consists of acid-insoluble proteins and the acquired pellicle, which can hinder effective acid etching (11,12).

Sodium hypochlorite is an excellent protein denaturant (11). Removal of excess proteins may enhance material adhesion to the tooth surface (12). The effects of enamel deproteinization using 5.25% sodium hypochlorite (NaOCl) prior to phosphoric acid (H3PO4) on the etching patterns as well as on the shear bond strength have been investigated previously (11-13). Espinosa *et al.* (12) reported that enamel deproteinization with 5.25% NaOCl for one min before H3PO4 etching improves the quality of enamel acid etching and therefore increases the enamel’s retentive surface significantly. In addition, Garrocho-Rangel *et al.* (9) demonstrated that 5.25% NaOCl deproteinization prior to enamel etching resulted in lower rates of microleakage between the enamel surface and the fissure sealant material than conventional H3PO4 etching alone. However, other studies concluded that deproteinization prior to or after etching did not change the topographic features of the enamel surface; thus, the use of 37% H3PO4 alone remains the best method for enamel pretreatment (11,13).

Recently, Pithon *et al.* (14) reported that enamel pretreatment with 10% papain gel increased the bond strength of orthodontic brackets. Papain, extracted from the latex of Carica papaya, is a proteolytic enzyme with antibacterial and anti-inflammatory properties. In 2003, a papain-based deproteinizing agent, Papacarie Duo® gel (Fórmula and Açäo, Brazil), was launched for the chemomechanical removal of caries. It contains chloramine, toluidine blue, salts, preservatives, stabilizers, thickener, and deionized water (15). Agarwal *et al.* (16) reported that enamel deproteinization with Papacarie® or 10% papain gel increased the shear bond strength of orthodontic brackets bonded to enamel when used before acid etching.

To the best of our knowledge, there are no published reports comparing the effect of 5.25% NaOCl and Papacarie® on the shear bond strength and the microleakage of fissure sealant materials. The null hypotheses of this study were 1) there is no effect on the shear bond strength of the fissure sealant after pre-treatment with either 5.25% NaOCl solution or Papacarie® gel; 2) deproteinization with 5.25% NaOCl solution or Papacarie® gel prior to acid etching does not decrease the microleakage between enamel surface and fissure sealant. Therefore, this *in vitro* study aimed to investigate and compare the effect of 5.25% NaOCl and Papacarie® on the shear bond strength of a fissure sealant when used prior to acid etching.

## Material and Methods

This study was approved by the ethical committee of Istanbul University Faculty of Dentistry (29.03.2018-No: 262). Written informed consent was also obtained from the study participants.

-Specimen preparation

One hundred and twenty-three freshly extracted human third molars were thoroughly washed in running water to remove blood, cleaned with a polishing paste without fluoride, and stored in sterile saline solution at room temperature until used for the study. Exclusion criteria were molars with visible fractures or cracks, carious lesions, enamel malformations, dental pathologies, or erosion.

**Figure 1:**
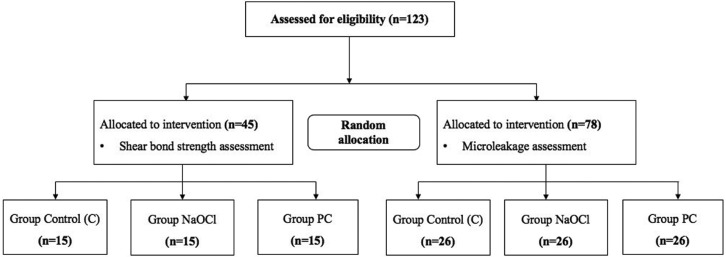
Flowchart presenting group assignment.

The sample size was calculated based on previous studies by Hatibovic-Kofman *et al.* (8) and Pithon *et al.* (14), utilizing G*Power 3.1.9.2 software. Using a power of 80% and significance level of 5%, a total sample size of 45 teeth for the shear bond strength and 78 teeth for the microleakage test were calculated to be sufficient. For each assesment, the teeth were randomly assigned to three groups according to the enamel pretreatments (Fig. 1).

-Enamel pretreatment

The specimens were treated as follows:

Group C (Control): The enamel surface was etched with 37% H3PO4 gel (3M/ESPE, St. Paul, MN, USA) applied with a microbrush for 15 seconds, rinsed with deionized water for 20 seconds, and air dried for 10 seconds.

Group NaOCl: The enamel surface was pretreated with 5.25% NaOCl (Wizard; Rehber Kimya, Istanbul, Turkey) applied with a sterile cotton pellet for 60 seconds, rinsed with sterile water, dried for 10 seconds, and etched as in Group C.

Group PC: The enamel surface was pretreated with Papacarie Duo® gel (Papacarie Duo®, F & A Pharmaceutical Laboratory Ltd, Sao Paulo, Brazil) for 60 seconds. The gel was washed with deionized water for 20 seconds and then air dried for 10 seconds. The treated surface was etched as in Group C.

-Assessment of shear bond strength

Teeth roots were removed from the crown by using a slow-speed diamond saw (Isomet, Buehler Ltd., Lake Bluff, IL, USA) under water-cooling, and subsequently, the crowns were sectioned longitudinally into two halves in the mesio-distal direction. Forty-five enamel samples were prepared and embedded into metal molds filled with acrylic resin (SC Self-Cure Acrylic, Imicryl®, Konya, Turkey). Each sample’s surface was ground with 600-grit silicon carbide paper under running water for 10 seconds to obtain a flat enamel surface.

Following the enamel pretreatment in each group, transparent silicon molds of 4 mm height and 3 mm diameter were positioned to the buccal or lingual/palatinal surface of the crown. The resin based fissure sealant (Fissurit® FX, VOCO, Germany) was inserted into the molds and polymerized for 20 seconds each. Then, the silicon mold was removed, leaving a sealant cylinder (4 mm × 3 mm) adhering to the enamel surface. After 24 hours of storage in deionized water at 37oC, the shear bond strength of all specimens was measured using a universal testing machine (Instron 3345, Instron Corp., Norwood, MA, USA) at a crosshead speed of 1 mm/min. Bond strength values were recorded as newtons (N) initially and then converted into megapascals (MPa).

The fractures were investigated under a stereoscopic microscope (SteREO Discovery.V20, Carl Zeiss, Gottingen, Germany) with 40x magnification to assess the failure types of the fractures, which were classified as follows.

Adhesive failure: Debonding at the interface between enamel and fissure sealant.

Cohesive failure: Failure in the body of the sealant or enamel.

Mixed failure: Failure involving both the interface and the material.

Failure patterns were independently examined by two investigators blinded to the pre-treatments. For any disagreement between the types of failures, a reassessment was performed by the investigators together. Bond failure sites were not statistically analyzed.

-Microleakage evaluation

Twenty-four hours before the experimental procedures, the teeth were removed from the sterile saline solution and stored in deionized water at 37°C. The enamel pretreatment and the sealant application on occlusal surfaces of the specimens took place as previously described. Specimens were then subjected to 500 thermal cycles between 5°C and 55°C with an exposure time of 20 seconds. Following thermocycling, the roots of the teeth were isolated with wax (Boxing Wax Sticks, Kerr Corporation, Romulus, MI, USA), and then two layers of nail polish were applied up to 1 mm from the sealant borders to prevent dye infiltration. The specimens were then immersed in 0.5% basic fuchsin solution (Merck, Biesterfeld International GmbH, Bavaria, Germany) at 37°C for 24 hours. Samples were rinsed with tap water for one minute to remove excess dye from the enamel surfaces. After drying, each sample was embedded in acrylic blocks and sectioned in the bucco-lingual direction, using a water-cooled diamond disc on a low-speed Isomet device (Buehler Ltd., USA) to provide two sections of each tooth. The dye penetration in each section was examined under a stereomicroscope (SteREO Discovery.V20, Carl Zeiss, Gottingen, Germany) at 20X magnification by one examiner blinded to the treatment the tooth had received. Dye penetration was scored according to the rankle scale: 0: no dye penetration; 1: dye penetration limited to the outer half of the sealant; 2: dye penetration extending to the inner half of the sealant; 3: dye penetration extending to the underlying fissure (17).

-Statistical analysis 

The statistical analysis was performed using the NCSS program (Number Cruncher Statistical System, Utah, USA). The differences in the shear bond strength among the groups were tested for significance by one-way analysis of variance (ANOVA). Moreover, the Tukey multiple-comparison test was used to identify the intergroup differences. The Kruskal-Wallis test was used to compare the microleakage scores among groups for statistically significant differences. The level of significance was set at 0.05.

## Results

**Figure 2:**
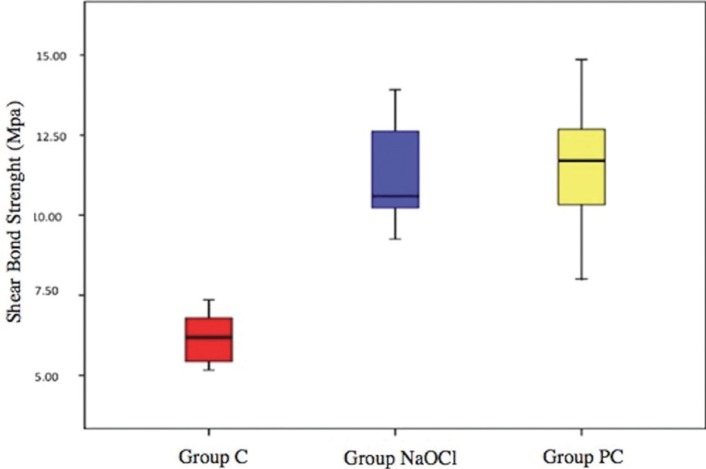
Shear bond strength values for each group.

The mean and standard deviation of the shear bond strength values for all groups are demonstrated in Table 1 and presented graphically in Figure 2. ANOVA showed the presence of significant differences (*p*=0.0001). Group PC exhibited the highest shear bond strength values (11.52 ± 1.87 Mpa), and Group C demonstrated the lowest (6.15 ± 0.72 Mpa). Tukey multiple-comparison test showed that the mean shear bond strength values of Group C were significantly lower than those of Group NaOCl and Group PC (*p*=0.0001). There were no significant differences between Group NaOCl and Group PC (*p*>0.05).

**Table 1:**
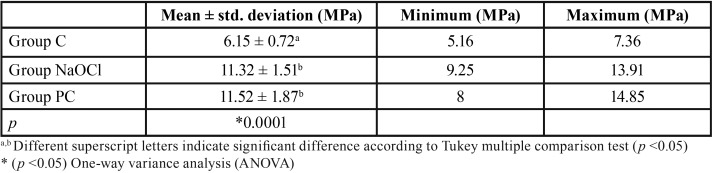
Mean shear bond strength values of the test groups (MPa).

Table 2 shows the fracture pattern analysis for all the debonded samples. The analysis of the bonding sites after the shear bond strength test revealed that adhesive failure was predominantly observed in all the groups. The most common failure types were adhesive failures (40-73.3%), followed by mixed failures (26.7-46.7%).

**Table 2:**
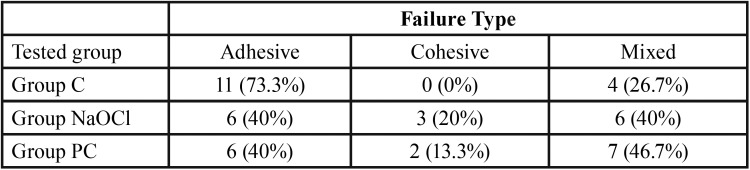
Failure types observed for each group.

**Figure 3:**
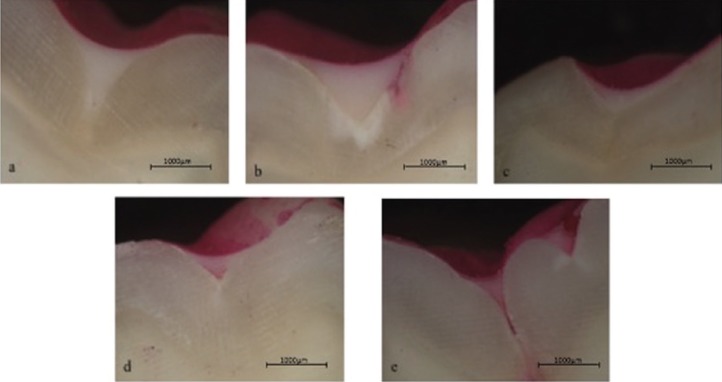
Stereomicroscopic images showing microleakage between the enamel surface and the fissure sealant (20X). (a) No dye penetration; (b) dye penetration up to one-half or less of the sealant depth; (c) dye penetration extending to the inner half of the sealant; (d) dye penetration to the sealant base; (e) dye penetration to the sealant base.

Table 3 shows the distribution of microleakage scores according to group. According to the Kruskal-Wallis test, no statistically significant difference was observed among the groups in terms of microleakage (*p*=0.863). The stereomicroscope images obtained from the samples can be seen in Figure 3.

**Table 3:**
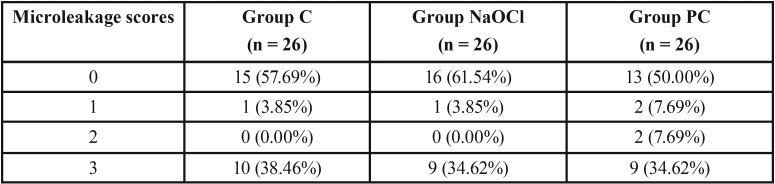
Distribution of the microleakage scores according to the groups.

## Discussion

Acid etching with H3PO4 does not necessarily result in a homogeneous and adequately etched surface, and this can lead to the eventual failure and loss of fissure sealant applications or restorations (12,18). The quantity and quality of etching are immensely important and depends on many factors such as the acid agent used, acid concentration, etching duration, and enamel surface composition (12). To increase etching quality and retention, both invasive and non-invasive techniques have been proposed and investigated. However, none of these techniques yielded favorable results (7,19).

Previous deproteinization studies have focused mainly on sodium hypochlorite because of its proteolytic activity and its well-known properties. However, it is a strong oxidizing agent and can affect the oral soft tissues if not used carefully, especially in young children. Due to the risk of adverse soft-tissue reactions and its poor smell and taste led to the search for an alternative material (11-13).

Papain was first introduced in dentistry in 2003 as a chemomechanical caries removal agent possessing promising properties Its use to remove partially degraded collagen in carious dentin has recently caused it to be investigated as a natural deproteinizing agent (15). Similar to sodium hypochlorite, it is a proteolytic agent (enzyme) and has antibacterial and anti-inflammatory properties (14,15). In previous studies, 10% papain gel (Papacarie) was used as a deproteinization agent (14,16). Therefore, in light of these studies, it was decided to use Papacarie®, a commercially available 10% papain gel, in the present study. Although enamel deproteinization with 5.25% NaOCl before acid etching increases the quality of the etching pattern, one must not forget that NaOCl is a strong oxidizing agent and may be disadvantageous to use in young, uncooperative children due to aspects such as poor taste and smell (12). Therefore, it may be beneficial to use a more natural deproteinizing agent, such as Papacarie® gel, which seems to be a promising alternative to sodium hypochlorite deproteinization.

In a previous study by Espinosa *et al.* (12), the effect of 5.25% sodium hypochlorite applied for 30 and 60 seconds prior to acid etching on the topographical features of the enamel surface was investigated by scanning electron microscopy (SEM) analysis, and it was reported that the quality of the etching pattern was superiorly enhanced after the 60-second application. Other studies have similarly used the deproteinization duration of 60 seconds (9,11,16,20,21). Therefore, in the present study, both deproteinizing agents were applied for 60 seconds.

Previous studies exhibited that enamel deproteinization with 5.25% NaOCl prior to H3PO4 etching increased the enamel’s retentive surface (12,22). Likewise, Valencia *et al.* (23) stated that the same technique improved Type I and II etch patterns by removing the organic materials from the enamel surfaces of teeth. According to Agarwal *et al.* (16), 10% papain gel or Papacarie® application prior to acid etching resulted in a better etching pattern and increased the shear bond strength of orthodontic brackets. Similarly, in the current study, the use of 5.25% NaOCl and Papacarie® gel increased the shear bond strength of the fissure sealant material significantly. Hence, the first null hypothesis of this study was rejected. This finding may be attributed to the fact that deproteinization prior to acid etching causes the removal of organic materials which are found in abundance in the interprismatic spaces. The etching process can affect both the center of the enamel prisms and the interprismatic spaces which in turn increases the shear bond strength of fissure sealants.

Few studies have investigated the effect of enamel deproteinization agents applied prior to acid etching and fissure sealant application in terms of microleakage (9,24). Garrocho-Rangel *et al.* (9) investigated the use of sodium hypochlorite as a deproteinization agent and its effect on the retention and microleakage of fissure sealants. They reported that although sodium hypochlorite deproteinization had no significant effect on retention, its application significantly reduced microleakage. In a study by Yamada *et al.* (24), artificial debris removal from pits and fissures by using the Carisolv system and sodium hypochlorite decreased the microleakage of the fissure sealant. No significant differences between the two agents were observed in their study. In contrast, in the present study, the application of sodium hypochlorite and Papacarie® as a deproteinization agent prior to etching did not decrease the microleakage of the fissure sealant. Although some previous studies have reported that fissure sealants with higher shear bond strength demonstrate less microleakage (25,26), the results of the present study demonstrated that both deproteinization groups possessed significantly higher shear bond strength values than the control group did, yet no statistically significant difference was determined between the groups in terms of microleakage. According to the findings of the current study, in terms of microleakage, no statistically significant difference was found between the deproteinization groups and the control group, which confirms the second null hypothesis.

Recently, the effect of Papacarie® gel on defective enamel surface has gained interest. It was reported that deproteinization with sodium hypochlorite and Papacarie® gel significantly increased the shear bond strength of only hypomineralized enamel and had no increasing effect on the shear bond strength with normal enamel (20). This finding differs from the current study, in which only healthy enamel was used. In light of the study by Ekambaram *et al.* (20), deproteinization could hold a positive future for obtaining improved fissure sealant adhesion to defective enamel surfaces.

The limitations of the current study were that the etching pattern of the enamel surface and the amount of the organic matter on the enamel surface were not evaluated. Also, hypomineralized teeth were not used in this study. If the effects of deproteinization agents on hypomineralized teeth containing high organic material had been evaluated, we may have been able to obtain beneficial results regarding the retention of restorative materials.

The results of the present study are preliminary findings to support the use of NaOCl and Papacarie® as an alternative deproteinization agents for the facilitation of increased adhesion and retention of fissure sealants. Further laboratory and clinical studies are necessary to investigate the retention of fissure sealants on both normal and defective enamel following enamel deproteinization.

## Conclusions

Under the conditions of this *in vitro* study, it was concluded that.

• Enamel deproteinization before acid etching increases the shear bond strength of the fissure sealant material.

• Deproteinization agents such as sodium hypchlorite and Papacarie® may be useful to help overcome adhesive restoration failures and improve fissure sealant applications.

• The application of deproteinization agents prior to etching did not decrease the microleakage of the fissure sealant.

• Papacarie® can be used as an alternative surface pre-treatment agent to sodium hypochlorite, which has many disadvantages, especially when working with pediatric patients.
